# Combined Parietal-Insular-Striatal Cortex Stroke with New-Onset Hallucinations: Supporting the Salience Network Model of Schizophrenia

**DOI:** 10.1155/2020/4262050

**Published:** 2020-01-22

**Authors:** Saheba Nanda, Krishna Priya, Tasmia Khan, Puja Patel, Heela Azizi, Deepa Nuthalapati, Christen Paul, Rabina Sippy, Abdulkader Hmidan Simsam, Jesslin Abraham, Gurjinder Singh, Alireza Goodarzi, Chiedozie Ojimba, Ayodeji Jolayemi

**Affiliations:** ^1^American University of Antigua College of Medicine, Department of Psychiatry, Interfaith Medical Center, Brooklyn, New York, USA; ^2^Department of Psychiatry, Interfaith Medical Center, Brooklyn, New York, USA; ^3^Medical University of the Americas, Department of Psychiatry, Interfaith Medical Center, Brooklyn, New York, USA; ^4^Medical University of Lublin, Department of Psychiatry, Interfaith Medical Center, Brooklyn, New York, USA

## Abstract

Brain imaging studies have identified multiple neuronal networks and circuits in the brain with altered functioning in patients with schizophrenia. These include the hippocampo-cerebello-cortical circuit, the prefrontal-thalamic-cerebellar circuit, functional integration in the bilateral caudate nucleus, and the salience network consisting of the insular cortex, parietal anterior cingulate cortex, and striatum, as well as limbic structures. Attributing psychotic symptoms to any of these networks in schizophrenia is confounded by the disruption of these networks in schizophrenic patients. Such attribution can be done with isolated dysfunction in any of these networks with concurrent psychotic symptoms. We present the case of a patient who presents with new-onset hallucinations and a stroke in brain regions similar to the salience network (insular cortex, parietal cortex, and striatum). The implication of these findings in isolating psychotic symptoms of the salience network is discussed.

## 1. Introduction

**Figure 1:**
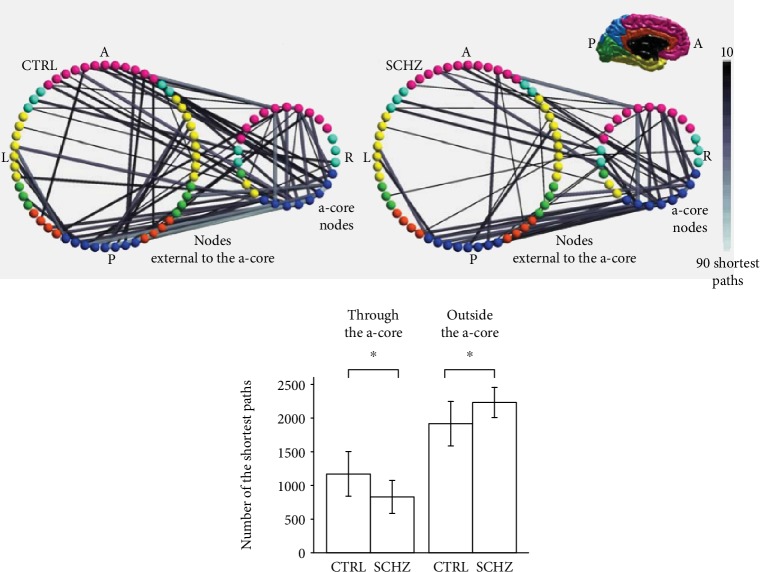
Reduced connections of brain areas in patients with schizophrenia [1].

As a result of rapid technological development in recent years, a range of functional imaging techniques are now available for the assessment of in vivo human brain function. Positron emission tomography (PET), single photon emission computed tomography (SPECT), and functional magnetic resonance imaging (fMRI) provide both temporal and spatial information that can be used to localize regional brain activity during the resting state or precisely controlled cognitive conditions. These techniques have considerably advanced our understanding of human brain function and the pathophysiology of schizophrenia. Magnetic resonance imaging (MRI) studies of patients with schizophrenia have robustly demonstrated local structural differences in multiple cortical areas, subcortical nuclei, and white matter tracts. An image published in the *Human Brain Mapping* journal depicts the wide range of disruptions and structural abnormalities; attributes to [1] are demonstrated in Figure 1.

The different abnormalities can be organized into brain circuits and networks that show functional and structural abnormalities. For instance, one of these circuits is the hippocampo-cerebello-cortical circuit involving the hippocampus, cerebellum, and occipital cortex. In the human brain, the basal ganglia connect distinct cortical areas and the cerebellum to specific thalamic areas [2, 3]. The limbic area, especially the hippocampus, displays intimate connections with the thalamus and the basal ganglia [4]. Thus, these brain regions, which display significant spatiotemporal functional consistency, can be organized as the hippocampo-cerebello-cortical circuit. In a study by Chen et al., a four-dimensional consistency of local neural activities (FOCA) was used to integrate temporal and spatial information comparing neural areas active in schizophrenia patients versus healthy controls. Compared with the healthy controls, schizophrenic patients exhibited increased local consistency in the hippocampus, basal ganglia, and cerebellar regions and decreased local consistency in the sensoriperceptual cortex. In addition to the aforementioned structural connections, causal effective functional connectivity was also observed in the hippocampo-cerebello-cortical (occipital) circuit. A Granger causal analysis (GCA) can measure this effective connectivity. A Granger causal connectivity from region A to another region B demonstrates how neuronal activity in A can predict the activity in B. Thus, GCA is a useful approach to identify the causal relationships that may exist between brain regions. These findings suggest that this circuit may play a role in motor dysfunction in schizophrenia [5].

Another abnormality is the prefrontal-thalamic-cerebellar circuit [1], which involves the medial prefrontal cortex (MPFC), the left and right thalamus, and the cerebellum. In schizophrenic patients, there is increased causal connectivity from the left thalamus to the MPFC when compared to healthy controls. In addition, there is also less causal connectivity from the right cerebellum to the left thalamus in schizophrenic patients compared to other psychiatric disorders such as depression [6]. The structural deficits in the MPFC and its causal connectivity from the cerebellum were associated with the negative symptom severity in patients with schizophrenia [6].

Another circuit of abnormal connection identified is the salience network (SN) consisting of the insular cortex, parietal anterior cingulate cortex, and striatum, as well as limbic structures [7]. While the posterior insula plays a key role in integrating sensory and motor information to mediate behavioral responses to interoceptive and external cues, the anterior portion of the insula functions to integrate this sensory and interoceptive feedback from the posterior insula with cognitive and emotional responses to the same stimuli, to create a conscious evaluation of affective experience. Clinically, insular dysfunction can plausibly account for several characteristic signs of psychoses such as deficits in social cognition, decision-making, information processing difficulties, and psychotic symptoms. Reduced insula–anterior cingulate cortex connectivity has been demonstrated in untreated patients with first-episode psychosis suggesting a normalization of this functional coupling via dopamine D2 receptor antagonism together with serotonin 2A receptor antagonism. A recent resting-state fMRI study showed that antipsychotic-induced improvement of psychotic symptoms was accompanied by increased functional connectivity among striatal regions, the anterior cingulate cortex, and the insula. In addition, the cortex of the right insula becomes significantly thinner in patients with schizophrenia. Chronic schizophrenia patients exhibited increased responses of aberrant salience in the striatum, hippocampus, and prefrontal regions as well as a lowered response of adaptive salience in the striatum, amygdala, hippocampus, and midbrain.

Attributing psychotic symptoms to any of these networks listed above is confounded by the presence of disruption of all these networks in schizophrenic patients. Such attribution can be done with isolated dysfunction in any of the aforementioned networks with concurrent psychotic symptoms. We present a patient with new-onset hallucinations and stroke in brain regions similar to the salience network, with the involvement of the insular and parietal regions and striatum. The implications of this in isolating psychotic symptoms of the salience network are further discussed.

## 2. Case Presentation

We present the case of a 72-year-old African American male who was brought in by family members to the psychiatric emergency department for new-onset auditory hallucinations that became increasingly worse. In the four months leading to the patient's presentation to the hospital, he had increasingly frequent and intense hallucinations as well as paranoid beliefs that usually occurred when he was driving. He believed people driving in other cars and pedestrians were trying to harm him. He began hearing congruent persecutory hallucinations while driving the car and made dangerous and strange driving maneuvers. These episodes of paranoia occurred in 15-20-minute intervals, lasted for a few minutes, and were accompanied by hallucinations. He was admitted to the inpatient psychiatric unit as he posed a danger to himself and others while in his state of psychosis.

The patient had a prior medical history that included hypertension and chronic kidney disease. He denied any past or current use of alcohol or other illicit substances. He had a prior psychiatric history in which he reported paranoid delusions since the age of 15. His paranoid beliefs centered around people in his family and arbitrary strangers he met who he believed were plotting to hurt him. The patient was on haloperidol 10 mg PO daily that managed his paranoid beliefs throughout his adult life. He worked successfully as paralegal, raised a family, and retired from work without any significant psychiatric episodes.

**Figure 2:**
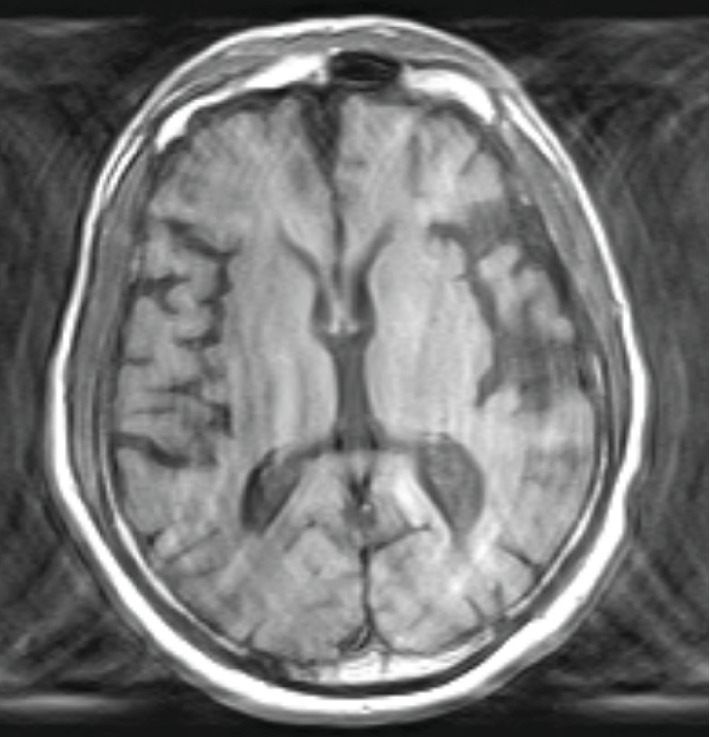
MRI of the brain showing the left parietal, insular, and lacunar infarct and atrophy.

Upon mental status examination, he exhibited significant psychomotor retardation; however, his gait was steady. His affect was blunted. His thought content was notable for paranoid beliefs. A cognitive assessment utilizing the Montreal Cognitive Assessment (MoCA) screening tool revealed a score of 20/30 while a MMSE revealed a score of 21/30 consistent with moderate cognitive impairment. Physical examination was within normal limits; however, he demonstrated decreased muscle strength ⅗ in both the right upper and lower extremities, along with absent reflexes in both the right biceps and triceps muscles. The results of laboratory and imaging findings were unremarkable as shown in Tables 1 and 2 and Figure 2.

Brain imaging is demonstrated in Figure 2.

An image of an MRI of the brain of the patient is shown in Figure 2. The MRI was compared with a prior CT scan two years before and indicates new pathology of a likely stroke involving the parietal cortex, insular cortex, and subcortical striatal structures and white matter/lacunar changes. There is no significant pathology of the hippocampus, cerebellum, occipital cortex, thalamus, or frontal corticosteroids. No acute cerebral cortical infarct or intracranial hemorrhage was present. Collateral information from medical records indicated a prior presentation in the emergency room for transient ischemic attack four and a half months prior to his presentation, and two weeks before the worsening of psychotic symptoms. He was managed with aspirin 81 mg and an outpatient follow-up.  Table 3 lists the MRI findings of the brain regions of interest.

Due to extrapyramidal symptoms, haloperidol was discontinued and sensitivity studies by gene therapy were performed for medications that could be started. The treatment plan included cognitive behavioral therapy with cognitive restructuring, supportive therapy, motor vehicle safety plan, and olanzapine 10 mg PO as needed. The patient showed significant improvement with psychological interventions and was discharged home with weekly outpatient cognitive behavioral therapy sessions.

## 3. Discussion

The case presented above demonstrated auditory hallucinations over a period of four months with escalating paranoid delusions; the symptoms were sudden and newly onset. The symptomatology is consistent with a late-onset psychosis that resembles the syndrome of schizophrenia. A review of the patient's past psychiatric history indicated no other period of his life for which he met criteria for schizophrenia. He was able to achieve good social and occupational functioning in his life. His age of presentation with this psychosis mimicking schizophrenia increases the likelihood of an underlying medical cause of this presentation. An extensive laboratory workup ruled out underlying acute metabolic or infective causes that could lead to the potential of delirium. Brain CT imaging revealed significant white matter changes consistent with stroke involving the parietal cortex, insular cortex, and subcortical striatal structures. These findings were consistent with medical records indicating a transient ischemic attack four and a half months prior to his admission and two weeks before the onset of hallucinations. There was no significant pathology of the hippocampus, cerebellum, occipital cortex, thalamus, or frontal cortices (Table 3). No acute cerebral cortical infarct or intracranial hemorrhage is present on imaging. The likely differential diagnosis given our patient's age includes psychosis due to general medical conditions such as the stroke that occurred two weeks prior to the emergence of hallucinations that involved the parietal cortex, insular cortex, and subcortical striatal structures of the brain.

The finding of the parietal lobe and insular and striatal regionsin this patient with white matter abnormalities is significant given the research on the attribution of schizophrenia symptoms to the different brain circuits. As stated above, in patients with schizophrenia, abnormalities have been found in circuits such as the hippocampo-cerebello-cortical circuit (involving the hippocampus, cerebellum, and occipital cortex), the prefrontal-thalamic-cerebellar circuit (which involves the medial prefrontal cortex, the left and right thalamus, and the cerebellum), and the salience network (consisting of the insular cortex, parietal anterior cingulate cortex, and striatum, as well as limbic structures). These abnormalities are demonstrated in most schizophrenic patients, and the attribution of symptoms to any of these networks has continued to become a subject of interest.

In our patient, there was no significant pathology of the hippocampus, cerebellum, and occipital cortex. Thus, it challenges the attribution of his auditory hallucinations and delusions to an abnormality in the hippocampo-cerebello-cortical circuit which is noted to be dysfunctional in patients with schizophrenia. In addition, there were no observed pathologies on brain imaging in this patient in the thalamus, prefrontal cortex, or cerebellum which also challenges the attribution of his auditory hallucinations and delusions to an abnormality in another circuit commonly dysfunctional in patients with schizophrenia called the prefrontal-thalamic-cerebellar circuit. This then leaves the potential role of the salience network which involves the insular cortex, parietal anterior cingulate cortex, and striatum, as well as limbic structures. The patient's brain CT revealed white matter changes consistent with stroke involving the parietal cortex, insular cortex, and subcortical striatal structures. The isolated pathology demonstrated in areas of the brain in this patient is similar to that in brain areas in the salience network in patients with schizophrenia. This suggests the possible association of hallucinations and delusions with the salience network.

**Figure 3:**
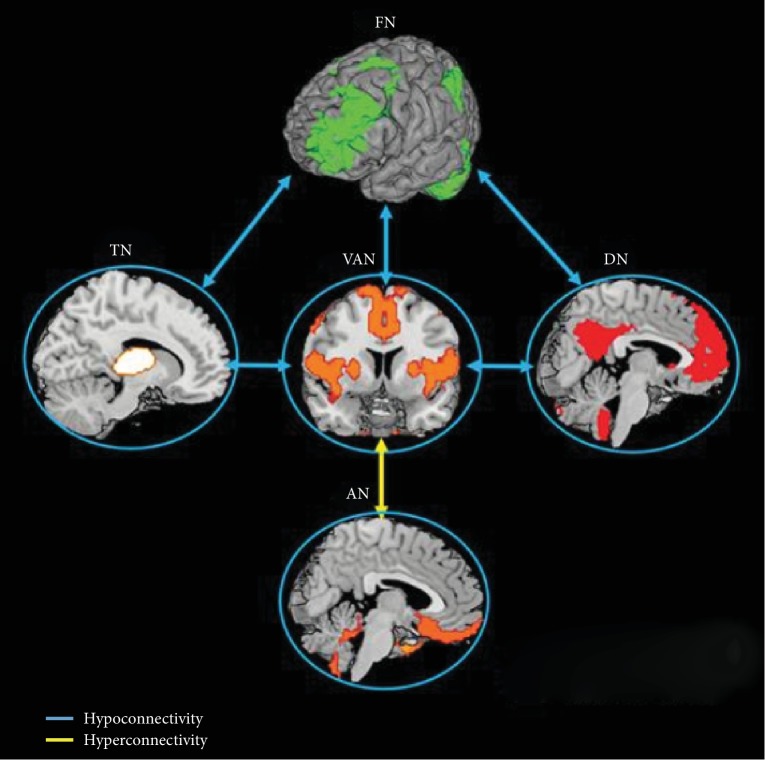
The salience network. Reduced connectivity within the salience monitoring systems (ventral attention network (VAN), thalamus network (TN)) and imbalanced connectivity among the salience systems and networks involved in internal thought (default network (DN)) and external goal-direction regulation (frontoparietal network (FN)) may reflect a weakness in salience processing that contributes to the general deficits in both external goal-directed behavior and self-awareness. Meanwhile, reduced connectivity between the FN and TN, which are involved in gathering information, may underlie the loss of salient information management control. Furthermore, decreased connectivity within neural systems involved in emotion processing and hyperconnectivity between the emotion system (AN) and the salience processing system (VAN) may relate to the deficits in emotion perception and regulation. Circles refer to reduced connectivity within the corresponding networks [7].

The salience network (insular cortex, parietal cortex, and striatum), shown in Figure 3, has been implicated in a function known as the salience monitoring and processing. Salience is defined as the effectiveness of a stimulus to stand out from its neighbors [7]. A stimulus might be considered to be salient by feature contrast, novelty, emotional, or motivational association. The integration of stimuli requires specific brain networks, which associate external stimuli with internal context, thus marking objects that require further consideration. Abnormal salience processing in patients with schizophrenic psychoses seems to arise from inappropriate evaluation of stimuli that would naturally be considered irrelevant. Hence, subthreshold stimuli become inappropriately attention-grabbing, which is then called aberrant salience. It is purported that abnormalities in salience processing could be the underlying cause of the inability of patients to control their internal thoughts and emotions, as well as their disconnection of self from the environment. This manifests in all patients exhibiting psychosis and psychotic-related disorders. In correlation with the model of schizophrenia, patients are unable to balance the activity of self and external stimuli in generating the appropriate response to given stimuli. Wang et al. [8] reported in one study that the extent of salience network disruption correlated with psychotic symptom severity and was more pronounced in subjects who developed psychosis, which can be utilized for the prediction of psychosis transition. In addition, parts of the salience network such as the insular cortex have been specifically associated with symptoms of hallucination. An atypical insula response is related directly with hallucinations and auditory hallucinations in schizophrenia correlate with an increase in hemodynamic response in the insula [9, 10]. People with schizophrenia have deficits similar to subjects with injuries in the insula and demonstrate abnormal insula response in tests that evaluate these functions. A negative correlation exists between the volume of grey matter in insula and positive symptoms in schizophrenia [11]. These findings suggest that there is a relationship between grey matter volume in the insula and a rise in hallucinations and delusions.

As mentioned above, this patient presents with sudden onset of auditory hallucinations over a period of four months, consistent with a late onset psychosis. A review of his past psychiatric history indicated no other period of his life for which he met the criteria for schizophrenia. Based on the patient's history, he may not have schizophrenia. The possibility of new-onset psychosis suggests the potential to speculate an association between psychotic symptoms and abnormalities in the salience network system involving the insular cortex, striatum, and parietal cortex. Other brain circuits involved in schizophrenia may not be necessary for the emergence of psychotic symptoms, even though they may play an important role in psychosis. Future studies may be needed to explore the symptomatology in a larger sample of patients with similar isolated abnormalities involving the insular cortex, striatum, and parietal cortex. In addition, one can explore studies of psychotic symptoms in patients with isolated strokes or other abnormalities of the hippocampo-cerebello-cortical circuit or the network of the thalamus and prefrontal cortex.

## 4. Conclusion

Multiple abnormal brain networks and circuits have been reported in patients with psychotic symptoms such as the hippocampo-cerebello-cortical circuit, the prefrontal-thalamic-cerebellar circuit, and the salience network consisting of insular cortex, anterior cingulate cortex, and striatum, as well as limbic structures. An isolated abnormality of the salience network could be sufficient for the emergence of hallucinations in patients. Future studies are needed to explore possible similar psychotic symptomatology in isolated abnormalities of the salience network, in order to better understand the neurobiology of positive symptoms of psychosis.

## Figures and Tables

**Table 1:** A complete blood count and comprehensive metabolic profile.

WBC	7.4	Sodium	140
RBC	5.18	Potassium	4.9
Hgb	15.9	Chloride	106
Hct	47.4	Carbon dioxide	26
MCV	91.5	Anion gap	8
MCH	30.7	BUN	21.0
MCHC	33.6	Creatinine	2.51
RDW	13.9	Kidney disease stage	26.99
Plt count	415 H	Glucose	109
MPV	8.7	Calcium	10.1
Neut %	54.9	Total bilirubin	0.4
Lymph %	29.2	AST	22
Mono %	6.8	ALT	49
Eos %	8.7	Alkaline phosphatase	182
Baso %	0.4	Troponin I	0.0
Neut %	4.0	Total protein	6.7
Lymph %	2.1	Albumin	4.2
Mono #	0.5		
Eos #	0.6		
Baso #	0.0		

**Table 2:** Urine toxicology, urine analysis, RPR, and other metabolic profiles.

Urine opiate screen	Negative	Albumin/globulin	1.7
Urine methadone	Negative	Lipase	16
Ur propoxyphene	Negative	TSH	0.922
Ur barbiturates	Negative	Free T4	1.4
Valproic acid	<4 L	Urine color	Yellow
Carbamazepine	<2.0 L	Urine clarity	Clear
Phencyclidine	Negative	Urine pH	6.0
Ur amphetamine	Negative	Ur specific gravity	1.015
Ur benzodiazepine	Negative	Urine protein	Negative
Lithium	<0.1 L	Urine glucose	Negative
Ur cocaine	Negative	Urine ketones	Small
Ur cannabinoids	Negative	Urine blood	Negative
Ethyl alcohol	<0 L	Urine nitrate	Negative
PT	11.8	Urine bilirubin	Negative
INR	1.04	Urine urobilinogen	0.2
APTT	32.2	Urine RUB	0-2 H
RPR titer/FTA	None	Urine WBC	1-5 H
		Ur Sq epithelial cells	Few H

**Table 3:** Gross pathology and volumetric characteristics of the different brain regions of the patient on MRI.

Brain regions	Volume and gross pathology comments	Reference range
Parietal cortex	Pathology consistent with stroke, total volume (13200 and 13000 cubic mm, respectively, right and left), superior parietal lobe volume loss (24080 cubic mm right and 23900 cubic mm left), posterior cingulate gyrus (28100 cubic mm right and 27800 cubic mm)	Total volume reference range 14500-14600 cubic mm, superior parietal lobe reference range 28085-28100 cubic mm, posterior cingulate gyrus (31400-31500 cubic mm)
Insular cortex	Pathology consistent with stroke, volume 4800 cubic mm right and 4690 cubic mm left	Reference range 7400-7600 cubic mm
Subcortical structures	Pathology consistent with ischemic changes in striatal structures and white matter/lacunar changes	
Frontal cortex	No volume loss in frontal cortex grey matter and no white matter changes, grey matter volume 133803 cubic mm right and 133811 cubic mm left	Reference range 133800-13400 cubic mm
Occipital cortex	No volume loss in occipital cortex grey matter and no white matter changes, grey matter volume 48788 cubic mm right and 48801 cubic mm left	Reference range 48700-48100 cubic mm
Temporal cortex	No volume loss in temporal cortex grey matter and no white matter changes, grey matter volume 127100 cubic mm right and 127125 cubic mm left	Reference range 127000-127200 cubic mm
Hippocampus	No volume loss (2900 cubic mm right and 2910 cubic mm left)	Reference range 2800-2950 cubic mm
Thalamus	No volume loss (7438 cubic mm right and 7390 cubic mm left)	Reference range 7300-7450 cubic mm
